# Genetic characterization of three recently discovered parvoviruses circulating in equines in China

**DOI:** 10.3389/fvets.2022.1033107

**Published:** 2022-12-08

**Authors:** JiaJun Ou, Jinghao Li, Xijie Wang, Lintao Zhong, Liang Xu, Jinxin Xie, Gang Lu, Shoujun Li

**Affiliations:** ^1^College of Veterinary Medicine, South China Agricultural University, Guangzhou, China; ^2^Guangdong Provincial Key Laboratory of Prevention and Control for Severe Clinical Animal Diseases, Guangzhou, China; ^3^Guangdong Technological Engineering Research Center for Pet, Guangzhou, China; ^4^Biological Disaster Prevention and Control, National Forestry and Grassland Administration, Shenyang, China; ^5^College of Veterinary Medicine, Xinjiang Agricultural University, Urumqi, China

**Keywords:** equine parvoviruses, genetic characterization, hepatitis, EqPV-CSF, EqCoPV, EqPV-H

## Abstract

The family *Parvoviridae* comprises many major viral pathogens that can infect humans and multiple other species, causing severe diseases. However, knowledge of parvoviruses that infect equids is limited. In the present study, we found that three equine parvoviruses (EqPVs), namely, equine parvovirus-hepatitis (EqPV-H), equine parvovirus-cerebrospinal fluid (EqPV-CSF) and equine copivirus (EqCoPV) cocirculated among horses in China. We examined the prevalence of these three EqPVs in 225 horse serum samples in China and found EqPV-H, EqPV-CSF and EqCoPV viremia in 7.6% (17/225), 2.7% (6/225) and 2.2% of samples (5/225), respectively. We also obtained the complete genomes of one EqPV-H strain, six EqPV-CSF strains and one EqCoPV strain. After phylogenetic analysis of the EqPVs, we found that EqPV-CSF and EqCoPV may have evolved from the same ancestor. The EqPV-CSF strains (E111 and A27) and EqCoPV strain (F124) were genetically similar to foreign strains, but the EqPV-CSF strains (B48, E96, C61 and F146) comprised unique clades. This study determined the prevalence of three EqPVs in Chinese horses and analyzed the genetic characteristics of EqPVs prevalent strains in Chinese horse herds. Our data provide a theoretical basis for follow-up research on the prevention and control of EqPVs.

## Introduction

*Parvoviridae* is a diverse family of nonenveloped and small single-stranded DNA viruses. It is divided into three subfamilies, *Densovirinae, Hamaparvovirinae* and *Parvovirinae*, which contain 11, 5 and 10 genera, respectively (https://talk.ictvonline.org/taxonomy/). The members of *Parvovirinae* can infect multiple species, causing severe diseases, as established in the early twentieth century (1). The majority of hosts can be infected by a variety of parvoviruses, resulting in diarrhea, abortion and other clinical features (2). For example, dogs can be infected with canine minute virus, canine bocavirus and canine bufavirus (3).

Equine parvovirus (EqPV) was first reported in 1985 in Canada; it was discovered in the liver and blood of an aborted equine fetus (4). Subsequent reports found that horses can be infected with a variety of EqPVs, including EqPV-hepatitis (EqPV-H), equine copivirus (EqCoPV) and EqPV-cerebrospinal fluid (EqPV-CSF), which vary in clinical signs and infection sites (5, 6). Among them, EqPV-CSF and EqCoPV were classified as belonging to the genus *Copiparvovirus*. EqPV-CSF was first reported in the CSF of a horse exhibiting neurological symptoms in the USA in 2015 (7). In 2018, China reported its first case of EqPV-CSF, which was found only in thoroughbred horses (8). In this same year, EqPV-H was first detected in the USA, in a horse that died from equine serum hepatitis (9). Subsequently, EqPV-H was detected in the USA, China, Germany, Brazil, South Korea and Austria; it is considered the viral agent associated with equine hepatitis (9–14). In 2019, a new type of EqPV was discovered in the USA in horse plasma through metagenomics technology and named EqCoPV (15). Related reports on EqCoPV in South Korea were published in 2021, with the research team detecting the presence of the virus in horse serum samples (14).

Although EqPVs are prevalent in horses in multiple countries, current genetic information on EqPV-H, EqPV-CSF and EqCoPV in the NCBI database is relatively limited. EqPV, belonging to *Parvoviridae*, is a small, nonenveloped DNA virus. Its genome has two open reading frames encoding nonstructural protein 1 (NS1) with a length of 566–594 amino acids and virion protein 1 (VP1) with a length of 975–1,078 amino acids. The pathogenicity of EqPVs remains unclear. However, despite serving as a potential pathogen of equine hepatitis, EqPVs have not attracted the attention of Chinese veterinarians (5). There are four reports that have investigated EqPVs in China: two reports concerning EqPV-H in Guangdong and one report concerning EqPV-CSF in Xinjiang; both from 2018 (8, 11, 16). In 2020, EqCoPV was also detected in Inner Mongolian horses. However, the co-infection of EqPVs and the characteristics and genetic evolution of EqPV strains are still unclear.

Here, we examined the prevalence and genetic history of three novel parvoviruses in horses in China. In this study, a total of 225 equine serum samples was collected from seven farms in Guangdong Province; these samples were used in polymerase chain reaction (PCR) assays to detect EqPV DNA. Detailed genetic and clinical analyses were conducted.

## Materials and methods

### Collection and preparation of samples

A total of 225 equine serum samples was collected from seven racetracks in Guangdong Province in 2019-2020, and the characteristics (sex, breed and age) of each horse were recorded. None of the horses that the samples were collected from were clinically apparent. All 200 μL samples were aliquoted into 1.5 mL EP tubes and stored at −80°C.

### DNA extraction, virus detection and screening

DNA from serum samples was extracted from 200 μL of the supernatant using a MiniBEST Viral RNA/DNA Extraction Kit (Takara, Japan) according to the manufacturer's instructions and then stored at −80°C until further use. To detect the presence of EqPVs, all samples underwent nested PCR using specific primers that targeted the NS1 gene. The primers EqPV-H-F1 and EqPV-H-R1 were used in the first round of PCR and EqPV-H-F2 and EqPV-H-R2 were used in the second round of PCR to detect EqPV-H; the primers EqPV-CSF-F and EqPV-CSF-R were used to detect EqPV-CSF; and the primers EqCoPV-F and EqCoPV-R were used to detect EqCoPV (Table 1). The target gene was amplified using 2×Taq PCR StarMix with Loading Dye (GeneStar, China) with the following parameters: 95°C for 5 min; 35 cycles of denaturation at 95°C for 30 s, annealing at 60°C for 30 s, and extension at 72°C for 1 min; and a final extension at 72°C for 5 min. For sequencing, a positive DNA band was purified from agarose gels using a FastPure® Gel DNA Extraction Mini Kit (Vazyme, Nanjing, China). The purified DNA was cloned into the pCloneEZ-TA vector (Clone Smarter, USA) and transformed into *Escherichia coli* (*E. coli*) DH5α-competent cells (Weidi, China). After culturing for 10 h, bacterial clones were identified by PCR, and positive *E. coli* clones were identified; the positive samples were sent out for direct Sanger sequencing (TianYi, Wuhan, China). BLAST analysis of the sequencing results of positive samples was conducted in the National Center for Biotechnology Information (NCBI) database (https://blast.ncbi.nlm.nih.gov/Blast.cgi).

**Table 1:** Primers used for virus detection and genome sequencing.

**Virus**	**Primer**	**Primer sequence (5′to 3′)**	**Target sequence size (bp)**
PCR for detection			
EqPV-H	EqPV-H-F1^a^	GGAGAAGAGCGCAACAAATGCA	453 bp
	EqPV-H-R1^b^	AAGACATTTCCGGCCGTGAC	
	EqPV-H-F2	GCGCAACAAATGCAGCGGTTCGA	580 bp
	EqPV-H-R2	GGCCGTGACGACGGTGATATC	
EqPV-CSF	EqPV-CSF-F	TAGCAGGAGCAATAGCAAGACAA	996 bp
	EqPV-CSF-R	ATTGGTGGTGGGACTATTTCTTC	
EqCoPV	EqCoPV-F	CAAATGGACCAATGCAAGCA	220 bp
	EqCoPV-R	ATGGTCCATAGTGACCAGTGTC	
PCR for genome sequencing			Target Region
EqCoPV	EqCoPV-F64-686	ATGACAGAGAGATTCTTCACA	64–686 bp
	EqCoPV-R64-686	GTTCCCTTACTCGTGTTCC	
	EqCoPV-F146-1232	AACTACATCGTATCCAGGTAC	146–1,232 bp
	EqCoPV-R146-1232	CTTGTAAGTGCTTTTGCAGCT	
	EqCoPV-F1208-2088	TGGAAGCTGCAAAAGCACT	1,208–2,088 bp
	EqCoPV-R1208-2088	TTTCTCCGTCCTCCCACCA	
	EqCoPV-F1910-2907	GTGGAGCCCCTCGCTGG	1,910–2,907 bp
	EqCoPV-R1910-2907	ACCCAAGATCATGAAGAGCAG	
	EqCoPV-F2821-3971	ACCAATACACAGGGCCAGGAA	2,821–3,971 bp
	EqCoPV-R2821-3971	CCATGGGTTTGCATTTCTTGT	
	EqCoPV-F3792-4696	TGGGCACCATACAAATGGGA	3,792–4,696 bp
	EqCoPV-R3792-4696	AATGGTGGTCTTCCTAATGTT	
	EqCoPV-F4578-5093	AAAGAAATAACATATGGGGAT	4,578–5,093 bp
	EqCoPV-R4578-5093	TTATTTGGTTTTACAATTCCT	
EqPV-CSF	CSF-1398F	GGACGACAGTGCATCATTATA	1–1,398 bp
	CSF-1398R	CCACTCTTTTAACAGTTCTGC	
	CSF-1629F	TACATAGACGACCAAGAATT	1,519–3,148 bp
	CSF-1629R	GTTAGTTGGCATTGTCTAGTTTGT	
	CSF-1937F	TGCCCTGAAAATGTTACGCAT	2,985–4,922 bp
	CSF-1937R	ACGCATATCCGGTGACG	
EqPV-H	EqPV-H-1104F	ATGGAGACCTTTTGGTACGG	1–1,104 bp
	EqPV-H-1104R	GGGAATGTCATTGAACGGGAA	
	EqPV-H-2508F	AGACGGGGAAACGTAATGCT	962–3,469 bp
	EqPV-H-2508R	CTACCACACCGACAGTTGTA/G	
	EqPV-H-1690F	TCAAACACGTCGCTGCATTC	3,054–4,743 bp
	EqPV-H-1690R	CAACACGATTTTATTGCATTACCGT	

a F, forward primer;

b R, reverse primer.

### Whole genome sequencing

The genome sequences of all EqPV-CSF, EqPV-H, and EqCoPV strains were downloaded from the NCBI database and compared in BioEdit 7.0.9.0. Multiple pairs of primers for the conserved region were designed to cover the complete genomes of EqPV-CSF, EqPV-H, and EqCoPV in Oligo 7.0 (Table 1). The targeting sequence was amplified using Phanta Max Super-Fidelity DNA Polymerase (Vazyme China) with the following PCR parameters: 95°C for 3 min; 35 cycles of denaturation at 95°C for 30 s, annealing at 60°C for 30 s, and extension at 72°C for 2 min; and a final extension at 72°C for 5 min. The positive DNA band was purified and cloned into pCloneEZ vectors (Clone Smarter, USA) and transformed into *E. coli* DH5α-competent cells (Weidi, China). After culturing for 10 h, bacterial clones were identified by PCR, and the positive *E. coli* clones were identified and sent for sequencing (TianYi, Wuhan, China). BLAST analysis of the sequencing results of positive samples was conducted in the NCBI database. Then, virus nucleotide sequences were assembled with SeqMan7.1.0.

### Sequence analysis and phylogenetic analyses

The nucleotide sequences of the positive samples were aligned using BioEdit 7.0.9.0, and the nucleotide identities were derived. Phylogenetic trees were established with MEGA 6.0 using the maximum likelihood method with the JTT+G model based on the bootstrap values of 1,000 replicates. A phylogenetic tree was constructed based on the published NS1 amino acid sequences of *Copiparvovirus* and all EqPV-CSF, EqPV-H, and EqCoPV strains.

## Results

### Virus detection in horses

In this study, we collected 225 equine serum samples from seven farms in Guangdong Province, including an equestrian training center (Supplementary Table 1). To investigate the positive rate of EqPVs in horses in China, we detected the presence of EqPV DNA in the serum samples. The average age of the tested animals was 9.1 years old (ranging from 1.5 to 25 years old), and the sample consisted of thoroughbreds (43, 19.1%), warmbloods (25, 11.1%), mixed breeds (136, 60.4%), ponies (2, 0.8%), Akhal-tekes (3, 1.3%), Mongolian horses (5, 3.6%) and Arabians (12, 5.3%). The tested horses included 49 stallions (21.8%), 88 mares (39.1%) and 88 geldings (39.1%) (Table 2).

**Table 2:** Characteristics of horses that serum samples were collected from in our study.

**Racetrack**	**Sample No**.	Sex^*^(M/S/G)	**Average age (range)^#^**	**Breed (horses)**	**EqPV-H**	**EqPV-CSF**	**EqCoPV**
A	31	4/19/8	6.1 (1.5–12)	Thoroughbred (8); Warmblood (1); Mixed-breed (22);	2 (6.5%)	1 (3.2%)	1 (3.2%)
B	28	12/16/0	6.2 (2–17)	Mixed-breed (28)	2 (7.1%)	1 (3.6%)	2 (7.1%)
C	18	8/7/3	7.7 (2–23)	Thoroughbred (1); Warmblood (2); Mixed-breed (15)	1 (5.6%)	1 (5.6%)	0 (0.0%)
D	14	2/3/9	9.6 (2–24)	Thoroughbred (5); Warmblood (2); Mixed-breed (5); Akhal-teke horse (1); Arabian horse (1)	1 (7.1%)	0 (0.0%)	0 (0.0%)
E	30	4/0/26	12.6 (2–25)	Thoroughbred (11); Warmblood (11); Mixed-breed (4); Arabian horse (4)	2 (6.7%)	2 (6.7%)	0 (0.0%)
F	51	7/23/21	10.1 (2–24)	Thoroughbred (5); Warmblood (5); Mixed-breed (35); Pony (2); Akhal-teke horse (1); Arabian horse (3)	5 (9.8%)	1 (2%)	2 (3.9%)
G	53	12/20/21	9.5 (2–24)	Thoroughbred (13); Warmblood (3); Mixed-breed (32); Akhal-teke horse (1); Arabian horse (4)	4 (7.5%)	0 (0.0%)	0 (0.0%)
Total	225	49/88/88	9.1 (1.5–25)	Thoroughbred (43); Warmblood (24); Mixed-breed (146); Pony (2); Akhal-teke horse (3); Arabian horse (12)	17(7.6%)	6(2.7%)	5(2.2%)

* M/S/G: male/stallion/gelding.

# Range: age range of horses in 2019.

After PCR and agarose gel electrophoresis analysis, a total of 24 equine serum samples presented a band with a size similar to the expected size of the EqPVs. Sequencing and online BLAST analysis showed that 17 samples were positive for EqPV-H DNA (7.6%), 6 samples were positive for EqPV-CSF (2.7%) and 5 samples were positive for EqCoPV (2.2%). These results demonstrated that three novel parvoviruses (EqPV-H, EqPV-CSF and EqCoPV) circulated in equine populations at these seven farms. However, all EqPV-positive horses were clinically healthy.

Detailed information on the equine samples positive for EqPV DNA is provided in Table 3. EqPV-H, EqPV-CSF and EqCoPV DNA were detected in horse serum samples at seven, four and three farms, respectively. Most of the EqPV-positive animals were mares (EqPV-H: 7/17, EqPV-CSF: 3/6 and EqCoPV: 2/5) and of mixed breed (20/24; EqPV-H: 13/17, EqPV-CSF: 4/6 and EqCoPV: 3/5), with ages ranging from 2 to 23 years old (EqPV-H: 2–23 years old, EqPV-CSF: 2–23 years old and EqCoPV: 3–12 years old). Interestingly, we found that two EqPV-H-positive animals were coinfected with EqPV-CSF or EqCoPV. However, no horses were coinfected with EqPV-CSF and EqCoPV or with the three EqPVs in this study.

**Table 3:** Characteristics of horses whose serum samples contained EqPV-H, EqPV-CSF and/or EqCoPV.

**ID**	**Farm**	**Age^#^**	Sex^*^	**Breed**	**EqPV-H**	**EqPV-CSF**	**EqCoPV**
			**(M/S/G)**				
A2	A	11	G	Thoroughbred	-	-	+
A16	A	3	S	Mixed breed	+	-	-
A27^a^	A	5	S	Mixed breed	+	+	-
B48	B	2	M	Mixed breed	-	+	-
B50^a^	B	3	M	Mixed breed	+	-	+
B58	B	6	S	Mixed breed	+	-	-
B59	B	5	S	Mixed breed	-	-	+
B61	B	8	S	Mixed breed	-	+	-
C71	C	2	M	Mixed breed	+	-	-
D90	D	2	M	Mixed breed	+	-	-
E96^a^	E	23	G	Warm blood	+	+	-
E111	E	13	G	Warm blood	-	+	-
E116	E	11	G	Thoroughbred	+	-	-
F124	F	12	G	Warm blood	-	-	+
F146	F	5	S	Mixed breed	-	+	-
F150	F	12	S	Mixed breed	+	-	-
F152^a^	F	12	S	Mixed breed	+	-	+
F155	F	2	M	Mixed breed	+	-	-
F159	F	2	M	Mixed breed	+	-	-
F168	F	17	S	Mixed breed	+	-	-
G182	G	2	M	Mixed breed	+	-	-
G196	G	12	G	Akhal-teke horse	+	-	-
G207	G	17	S	Mixed breed	+	-	-
G216	G	12	G	Thoroughbred	+	-	-
Total	-	-	-	-	17	6	5

* M/S/G: male/stallion/gelding.

# Age (years) in 2019.

a Coinfected with two EqPVs.

### Viral genomic sequencing and analysis

To date, the whole genomes of a total of 16 EqPV-H strains, 3 EqPV-CSF strains and 4 EqCoPV strains have been reported. To better understand the genetic characterization of the three common EqPVs in China, we amplified the nearly complete viral genome of EqPVs with gap-filling PCR (Table 1). All genome sequencing primers were designed according to the complete EqPV genome sequences published in the NCBI database. After sequencing and assembly, we obtained the complete genomes of one EqPV-H strain (sample ID: B58), six EqPV-CSF strains (samples ID: A27, B48, C61, E96, E111 and F146), and one EqCoPV strain (sample ID: F124). The genome sequences have been submitted to the GenBank database (accession nos. OM31076-OM310771 and OM310773).

The EqPV-H genome is 5,308 nucleotides long and contains NS1 (1,779 nucleotides), intergenic region 1, VP1 (2,922 nucleotides) and intergenic region 2. After sequence alignment and analysis of American, Korean, and Chinese strains, we found nucleotide identities of 95.8–99.9% and 95.2–100% for NS1 and VP1, respectively, among EqPV-H strains (Supplementary Table 2). Amino acid identities of 96.1–100% and 95.4–100% were found for NS1 and VP1, respectively, among EqPV-H strains. The nucleotides and amino acids of the NS1 and VP1 genes of the B58 strain were similar to those of the previously reported Chinese strains D14 and H40.

EqPV-CSF is a newly discovered EqPV; thus, genome sequences of its strains are relatively scarce, and only 3 strains in the NCBI database have relatively complete genomes. In this study, we successfully obtained nearly complete genomes (missing the first 155 nucleotides of the NS1 gene) of 6 EqPV-CSF strains with PCR. After sequence alignment and analysis, we found that the GC contents of the six EqPV-CSF strains were in the range of 42.30–42.71%. When these strains were compared with previously reported EqPV-CSF strains in the USA and South Korea, no nucleotide insertions or deletions were found. At the nucleotide level, six EqPV-CSF strains had similarities of 92.9–99.6% and 92.2–98.9% in the NS1 and VP1 genes, respectively. Compared to other EqPV-CSF strains, these six strains had similarities of 83.2–99.5% and 91.9–95.8% of the NS1 and VP1 genes, respectively (Table 4). At the amino-acid level, these six EqPV-CSF strains had similarities of 97.2–99.6% and 94.1–97.9% in NS1 and VP1, respectively, and similarities of 88.7–99.5% and 94.0–96.0% to previously reported strains. Interestingly, the nucleotide similarity between the NS1 gene of strain E111 and the USA strain in 2014 was 99.5%, and the similarities of the NS1 gene of the other strains in this study with the USA strain were 94.0–96.7%.

**Table 4:** Analysis of the nucleotide (upper right)/amino acid (bottom left) identity of the NS1 and VP1-coding sequences among EqPV-CSF strains.

	**S52^b^**	**Ky1^a^**	**USA 2014^a^**	**A27**	**B48**	**C61**	**E96**	**E111**	**F146**	**G+C content**
**The nucleotide (upper right)/amino acid (bottom left) identity of the NS1-coding sequence**										
S52^b^	***	97.6	84.5	84.1	83.7	84.1	83.2	84.2	84.1	38.81%
Ky1^a^	99.3	***	86.0	84.6	84.3	84.3	83.8	85.6	85.3	38.63%
USA 2014^a^	90.3	89.9	***	95.1	94.0	94.5	94.0	99.5	96.7	38.33%
A27	89.9	89.2	98.9	***	92.9	93.9	93.1	94.7	94.1	37.87%
B48	89.2	88.8	98.2	97.7	***	93.8	94.4	93.7	93.4	37.74%
S52^b^	89.0	88.7	97.7	97.7	97.3	***	96.2	94.4	97.6	38.91%
E96	88.8	88.5	97.9	97.5	98.4	97.9	***	93.9	94.0	38.33%
E111	89.7	89.4	99.5	98.4	97.7	97.2	97.5	***	96.4	38.33%
F146	88.7	88.7	97.7	97.3	97.3	99.6	97.9	97.2	***	38.98%
**The nucleotide (upper right)/amino acid (bottom left) identity of the VP1-coding sequence**										
S52^b^	***	96.0	95.2	93.6	93.6	92.9	93.4	93.4	93.4	44.71%
Ky1^a^	96.6	***	96.4	92.8	94.5	91.9	94.1	94.2	94.1	44.43%
USA 2014^a^	96.6	96.8	***	93.5	95.8	92.7	95.8	95.5	95.7	44.40%
A27	96.5	95.2	95.6	***	93.4	98.1	93.4	92.8	93.3	44.43%
B48	95.3	95.0	96.0	96.1	***	92.6	96.6	98.8	96.6	44.56%
C61	95.2	93.7	94.2	97.2	94.6	***	92.6	92.2	92.6	44.53%
E96	95.1	94.4	95.5	96.2	96.8	94.5	***	96.0	98.9	44.47%
E111	94.8	94.6	95.7	95.1	97.9	94.1	96.0	***	96.1	44.77%
F146	94.6	94.0	95.2	95.8	96.5	94.2	97.6	95.7	***	44.50%

a EqPV-CSF strains isolated from the USA;

b EqPV-CSF strains isolated from Korea.

Similar to EqPV-CSF, sequence information for EqCoPV in the NCBI database is relatively scarce; there are only five complete genome sequences of EqCoPV. Our study is the first to obtain complete NS1 and VP1 sequences for the EqCoPV strain in China (Table 5). After sequence alignment and analysis, we found that the EqCoPV F124 strain, similar to EqCoPV 8, has 3 nucleotides (ACA or AGC) deleted at positions 1,354-1,356 of the VP1 gene. Compared with reported strains, the F124 strain had similarities of 92.1–92.6% and 92.5–92.9% in the NS1 and VP1 genes, respectively, to other strains at the nucleotide level. At the amino-acid level, the F124 strain had similarities of 94.0–95.9% and 95.5–97.3% in the NS1 and VP1 genes, respectively, to the EqCoPV 8, 9, 11 S109 and A4 strains. Moreover, the F124 strain was more similar to the USA strain than to the South Korean and Chinese strains.

**Table 5:** Analysis of the nucleotide (upper right)/amino acid (bottom left) identity of the NS1 and VP1-coding sequences among EqCoPV strains.

	**EqCoPV 8^a^**	**EqCoPV 9^a^**	**EqCoPV 11^a^**	**EqCoPV S109^b^**	**EqCoPV F124^c^**	**EqCoPV A4^c^**	**G+C content**
**The nucleotide (upper right)/amino acid (bottom left) identity of the NS1-coding sequence**							
EqCoPV 8^a^	***	95.6	99.6	92.3	93.1	92.1	42.45
EqCoPV 9^a^	96.1	***	95.5	92.1	94.8	92.6	43.08
EqCoPV 11^a^	100.0	96.1	***	92.4	93.2	92.1	42.28
EqCoPV S109^b^	95.2	95.0	95.2	***	90.9	92.2	42.45
EqCoPV A4^c^	92.5	94.7	92.5	92.6	***	92.4	43.19
EqCoPV F124^c^	94.3	95.2	94.3	95.9	94.0	***	41.94
**The nucleotide (upper right)/amino acid (bottom left) identity of the VP1-coding sequence**							
EqCoPV 8^a^	***	96.4	99.1	92.3	93.7	92.9	45.63
EqCoPV 9^a^	97.9	***	96.2	92.3	94.0	92.5	45.93
EqCoPV 11^a^	98.5	97.5	***	92.1	93.2	92.6	45.62
EqCoPV S109^b^	97.0	96.5	96.4	***	92.1	92.5	46.31
EqCoPV A4^c^	96.6	96.8	95.8	96.0	***	92.7	45.98
EqCoPV F124^c^	97.3	96.4	96.7	97.2	95.5	***	45.88

a EqCoPV strains isolated from the USA;

b EqCoPV strains isolated from Korea;

c EqCoPV strains isolated from China.

### Phylogenetic analysis

**Figure 1:**
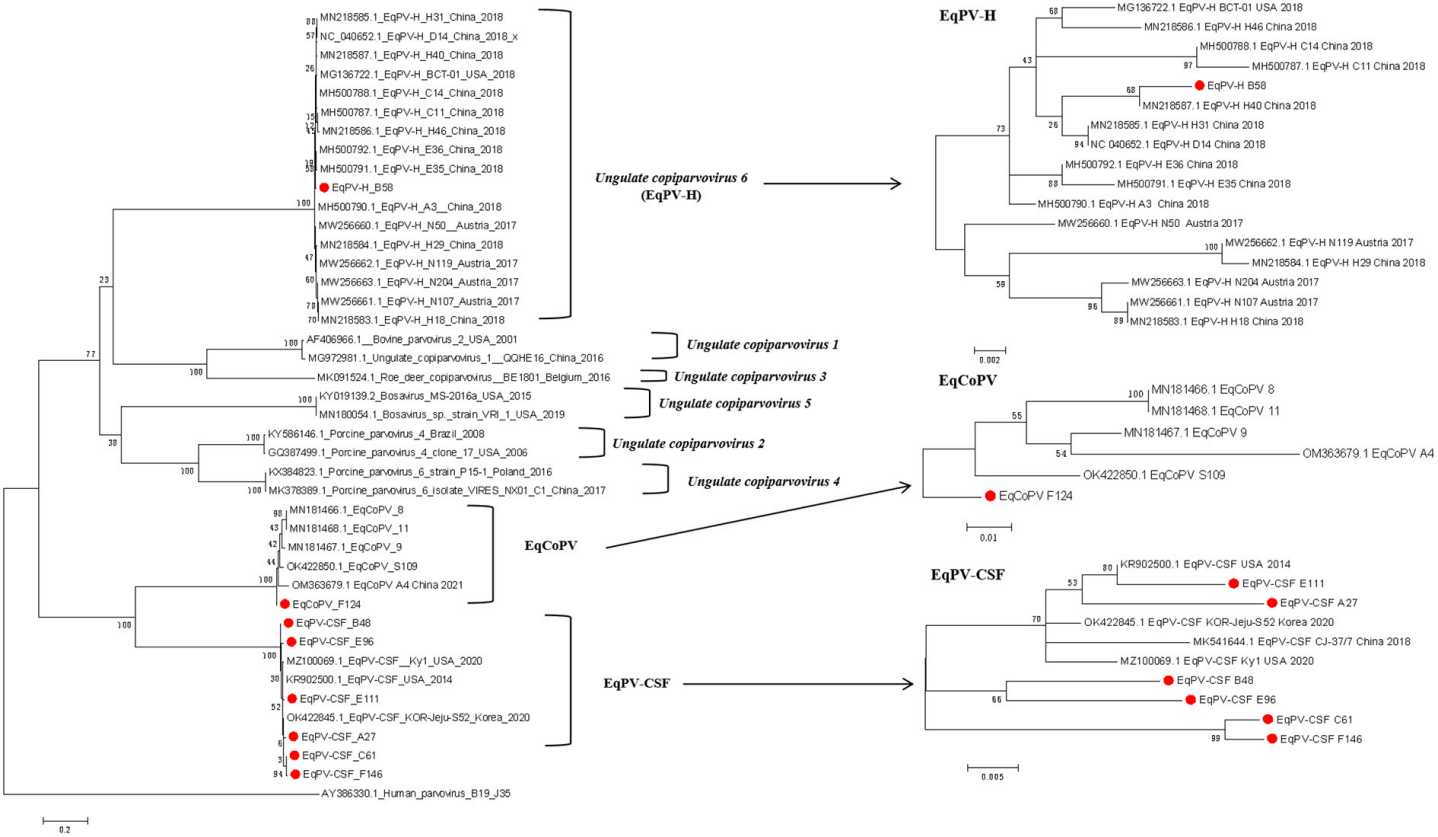
Phylogenetic analysis based on complete NS1 amino acid sequences of the genus *Copiparvovirus*. Chinese The Chinese strains isolated in this study are indicated by circles filled in red.

To identify the origin of these viruses, we conducted phylogenetic analyses of *Copiparvovirus* (Figure 1). We found that the EqPV-H strains grouped together, and that EqPV-CSF and EqCoPV grouped together, indicating that EqPV-CSF and EqCoPV likely evolved from the same ancestor. In addition, the EqPV-H strains isolated in this study were grouped with other Chinese strains, and the EqCoPV strains (F124) isolated in this study were grouped with a separate clade. The EqPV-CSF isolates from this study were grouped with the USA strains or divided into two branches (B48 with E96 and C61 with F146).

## Discussion

EqPVs were first reported in 1985; they have since been reported in many countries. Moreover, these viruses can be transmitted through serum products, infecting horses and resulting in hepatitis (9, 17). As EqPVs are potential pathogens, their genetic diversity should be monitored and observed. In this study, three newly identified EqPVs, EqPV-H, EqPV-CSF and EqCoPV, were observed in horses in China. Moreover, we obtained complete genomes for 1 EqPV-H strain, 6 EqPV-CSF strains and 1 EqCoPV strain by gap-filling PCR, sequencing, assembly and alignment.

As expected, we detected EqPV DNA in Chinese horses. The prevalence rates of EqPV-H, EqPV-CSF and EqCoPV in the horses sampled in this study were 7.6% (17/225), 2.7% (6/225) and 2.2% (5/225), respectively, as determined with PCR. The prevalence of EqPV-H was similar to that previously reported (11.9%), while the prevalence of EqPV-CSF was lower than that previously reported in Xinjiang (25%) (8, 16). Previous studies have indicated that EqPVs are blood-borne viruses transmitted between horses through contaminated horse blood products. However, after we consulted veterinarians at these farms, we found that none of the EqPV-positive horses in this study had been injected with commercial horse serum or related products. Moreover, we found that preventative measures against the spread of these blood-borne viruses were relatively weak at these farms. For example, many mosquitoes are found near horse farms, and methods of disinfection during administration of injections are not standardized. The routes of EqPV transmission have been speculated to include blood-borne vectors and contaminated medical equipment (9). This study only detected the presence of EqPV DNA in horse serum, but it remains unclear whether this DNA was protected in capsids (and thus potentially infectious) or merely pieces of viral sequences circulating in the blood without an actual infection. Therefore, the detailed mechanism of transmission of EqPVs needs further study.

We found horses that were coinfected with EqPV-H and EqPV-CSF or with EqPV-H and EqCoPV but no horses coinfected with EqCoPV and EqPV-CSF or with three EqPVs. Interestingly, this result is similar to our previous study on EqPV-H, equine hepacivirus (EqHV), equine pegivirus (EPgV), and Theiler's disease-associated virus (TDAV), which found coinfection of EqPV-H and other blood-borne viruses (11). Furthermore, the present study found a high rate of EqHV coinfection in horses with EqPV-H viremia (9/17, 52.9%).

In the phylogenetic tree analyses, the Chinese EqPV-CSF and EqCoPV strains were highly similar to strains isolated from the USA and South Korea, while EqPV-H was placed in its own clade. This finding indicates that both EqPV-CSF and EqCoPV evolved from a common ancestor. More importantly, we found that not only were the Chinese EqPV-CSF (E111 and A27) strains and an EqCoPV (F124) strain genetically similar to foreign strains but the EqPV-CSF strains (B48, E96, C61 and F146) were comprised of unique clades. EqPV-CSF was reported in China in 2018; the report presented valuable information showing that EqPV-CSF-like virus was likely exotic and introduced into Xinjiang through international horse trade. However, through our phylogenetic analysis, we found that the EqPV-CSF (B48, E96, C61 and F146) strains were divided into independent clades. This suggests that EqPV-CSF may have been circulating in China prior to 2018 and evolved into a separate branch. Previous studies have found that there is a phenomenon of strain recombination in EqPV-H (11, 18). However, we did not find recombination in the isolates in this study through the Recombination Detection Program (RDP) version 4.27.

In conclusion, this is the first report to identify and describe the genetic characteristics of three novel EqPVs in horses in China; we found that Chinese horses are exposed to multiple EqPVs. We also amplified the complete genome sequences of 8 EqPVs to facilitate future studies on the epidemiological characteristics, clinical significance, genetic diversity and evolution of EqPVs.

## Data availability statement

The datasets presented in this study can be found in online repositories. The names of the repository/repositories and accession number(s) can be found in the article/Supplementary material.

## Ethics statement

No studies involving human participants or animals performed by any of the authors are described in this article. This study uses 225 samples obtained from racetracks in Guangdong province. The South China Agricultural University Experimental Animal Welfare Ethics Committee did not require the study to be reviewed or approved by an ethics committee because all the samples in this study originate from diagnostic samples. Written informed consent was obtained from the owners for the participation of their animals in this study.

## Author contributions

JO performed the experiments and analyzed the data. XW, JL, JX, GL, and SL participated in whole study design, coordinated the study, and prepared the manuscript and manuscript revision. LX and LZ assisted with conducting the experiments. All authors have read and agreed to the published version of the manuscript.
