# Supplementary material for: Genetic characterization of three recently discovered parvoviruses circulating in equines in China
Source: Front Vet Sci. 2022 Dec 8;9:1033107. doi: 10.3389/fvets.2022.1033107 (PMC9773246; doi:10.3389/fvets.2022.1033107)
Supplement: Supplementary file 2 [file Table_2.DOCX]

[Supplementary Table 2](https://www.frontiersin.org/articles/10.3389/fvets.2020.00121/full#SM1)

Analysis of the nucleotide (upper right)/amino acid (Bottom left) identity of the NS-coding sequence between EqPV-H strains

|  | BCT-01^a^ | B58 | N107^B^ | N204^B^ | N50^B^ | N119^B^ | A3^C^ | C14^C^ | C11^C^ | H40^C^ | H46^C^ | H31^C^ | H29^C^ | H18^C^ | D14^C^ | E36^C^ | E35^C^ |
| --- | --- | --- | --- | --- | --- | --- | --- | --- | --- | --- | --- | --- | --- | --- | --- | --- | --- |
| BCT-01a | *** | 98.8 | 97.8 | 97.8 | 97.8 | 97.5 | 98.5 | 97.6 | 97.3 | 98.9 | 98.8 | 99.0 | 97.6 | 97.9 | 99.0 | 98.8 | 98.9 |
| B58 | 98.8 | *** | 97.5 | 97.5 | 97.7 | 97.4 | 98.8 | 97.5 | 97.0 | 99.7 | 98.3 | 99.3 | 97.5 | 97.6 | 99.3 | 98.9 | 98.9 |
| N107B | 98.1 | 98.0 | *** | 99.6 | 97.0 | 97.1 | 97.3 | 96.5 | 96.2 | 97.5 | 97.2 | 97.6 | 97.0 | 99.8 | 97.6 | 97.5 | 97.6 |
| N204B | 98.1 | 98.0 | 99.7 | *** | 97.1 | 97.1 | 97.3 | 96.7 | 96.4 | 97.6 | 97.4 | 97.7 | 97.0 | 99.8 | 97.7 | 97.6 | 97.6 |
| N50B | 98.1 | 98.3 | 98.5 | 98.5 | *** | 98.1 | 97.6 | 97.2 | 97.0 | 97.5 | 97.3 | 97.7 | 98.2 | 97.2 | 97.6 | 97.8 | 97.8 |
| N119B | 97.1 | 97.3 | 98.0 | 98.0 | 98.1 | *** | 97.4 | 96.1 | 95.8 | 97.3 | 97.1 | 97.4 | 99.3 | 97.3 | 97.4 | 97.6 | 97.6 |
| A3C | 99.2 | 99.3 | 98.3 | 98.3 | 99.0 | 97.6 | *** | 97.4 | 97.0 | 98.5 | 98.0 | 98.7 | 97.4 | 97.4 | 98.7 | 98.8 | 98.9 |
| C14C | 98.5 | 98.3 | 97.6 | 98.0 | 97.6 | 96.6 | 98.7 | *** | 99.2 | 97.6 | 97.0 | 97.8 | 96.1 | 96.7 | 97.8 | 97.6 | 97.8 |
| C11C | 98.1 | 98.0 | 97.3 | 97.6 | 97.3 | 96.3 | 98.3 | 99.7 | *** | 97.0 | 97.4 | 97.1 | 95.8 | 96.4 | 97.1 | 97.1 | 97.3 |
| H40C | 98.8 | 99.7 | 98.0 | 98.0 | 98.0 | 97.0 | 99.0 | 98.3 | 98.0 | *** | 98.3 | 99.6 | 97.4 | 97.7 | 99.6 | 98.7 | 98.8 |
| H46C | 99.2 | 98.7 | 98.0 | 98.0 | 98.0 | 97.0 | 99.0 | 98.3 | 98.7 | 98.7 | *** | 98.5 | 97.3 | 97.4 | 98.4 | 98.3 | 98.3 |
| H31C | 99.2 | 99.0 | 98.3 | 98.3 | 98.3 | 97.3 | 99.3 | 98.7 | 98.3 | 99.3 | 99.0 | *** | 97.5 | 97.8 | 99.9 | 98.9 | 98.9 |
| H29C | 97.0 | 97.1 | 97.8 | 97.8 | 98.0 | 99.8 | 97.5 | 96.5 | 96.1 | 96.8 | 96.8 | 97.1 | *** | 97.1 | 97.5 | 97.6 | 97.6 |
| H18C | 98.1 | 98.0 | 100.0 | 99.7 | 98.5 | 98.0 | 98.3 | 97.6 | 97.3 | 98.0 | 98.0 | 98.3 | 97.8 | *** | 97.8 | 97.7 | 97.8 |
| D14C | 99.2 | 99.0 | 98.3 | 98.3 | 98.3 | 97.3 | 99.3 | 98.7 | 98.3 | 99.3 | 99.0 | 100.0 | 97.1 | 98.3 | *** | 98.8 | 98.9 |
| E36C | 99.0 | 99.2 | 98.3 | 98.3 | 98.7 | 97.6 | 99.5 | 98.5 | 98.1 | 98.8 | 98.8 | 99.2 | 97.5 | 98.3 | 99.2 | *** | 99.7 |
| E35C | 98.7 | 98.8 | 98.0 | 98.0 | 98.3 | 97.3 | 99.2 | 98.5 | 98.1 | 98.5 | 98.5 | 98.8 | 97.1 | 98.0 | 98.8 | 99.7 | *** |

^A^: EqPV-H strains isolate from USA; ^B^: EqPV-H strains isolate from Austria; ^C^: previous EqPV-H strains isolate from China.
